# Predicting treatment-related cardiovascular risks in breast cancer patients: development and validation of an interpretable machine learning model

**DOI:** 10.3389/fonc.2026.1814219

**Published:** 2026-08-12

**Authors:** Luxin Wang, Rui Yan, Xinyu Zhu, Jinming Yu, Fangfang Cui

**Affiliations:** 1 Key Laboratory of Public Health Safety, Ministry of Education, School of Public Health, Fudan University, Shanghai, China; 2 Internet Medical and System Applications of National Engineering Laboratory, The First Affiliated Hospital of Zhengzhou University, Zhengzhou, Henan, China

**Keywords:** breast cancer, cardio-oncology, cardiovascular risk, electronic medical records, machine learning

## Abstract

**Purpose:**

This study aimed to develop and validate an interpretable machine learning model to predict the 1- to 3-year risk of cardiovascular events in breast cancer patients by integrating baseline and treatment variables, while preliminarily investigating the potential association between short-term cardiac function decline and long-term adverse cardiovascular events.

**Methods:**

We analyzed electronic medical records from 31,878 breast cancer patients. A composite cardiovascular event outcome was used. Predictors were selected via a two-step process: removing highly correlated variables (|r|≥0.7) and applying LASSO regression with 10-fold cross-validation, which refined 62 initial variables down to 18. Five models were built and compared using the area under the receiver operating characteristic curve (AUC-ROC). The optimal model was interpreted using SHapley Additive exPlanations (SHAP).

**Results:**

Among 31,878 breast cancer patients, 3,960 (12.4%) experienced cardiovascular events. The XGBoost model demonstrated the best overall discriminative performance (AUC = 0.790). SHAP analysis identified endocrine therapy, anemia management therapy, and history of cerebrovascular disease as the top three predictors. Crucially, short-term decline in cardiac function was also selected as a significant predictor, supporting its role as a precursor to long-term events. Model robustness was confirmed via sensitivity analysis.

## Introduction

Breast cancer remains a major global health challenge and the most frequently diagnosed malignancy in women worldwide. According to the 2022 Global Cancer Statistics, it ranks second in incidence and fourth in mortality among female cancers (1). The situation is particularly concerning in China, where breast cancer represents the second most common cancer in women, with an observed trend toward earlier onset (2). While advances in early detection and treatment have shifted breast cancer management toward chronic care, a significant challenge has emerged: 2%–15% of survivors develop cardiovascular disease, now the leading non-cancer cause of death in this population (3, 4). Recent systematic reviews have demonstrated that cardiovascular disease is a major competing cause of death among breast cancer survivors. Particularly for those exposed to cardiotoxic treatments or with pre-existing cardiovascular risk factors, the cardiovascular-specific mortality risk is significantly elevated compared to the general population (5). This elevated cardiovascular risk is closely linked to cancer therapy-related cardiovascular toxicity (CTR-CVT). As defined in the 2022 ESC guidelines on cardio-oncology, CTR-CVT encompasses a spectrum of cardiovascular injuries, including cancer therapy-related cardiac dysfunction (CTRCD), cardiomyopathy, myocarditis, hypertension, and arrhythmias, directly or indirectly induced by anticancer treatments (6). Specifically in breast cancer, established therapies such as chemotherapy, targeted therapy, endocrine therapy, and radiotherapy are all known to induce varying degrees of cardiotoxicity (7, 8), increasing the risk of cardiovascular adverse events during or after treatment. Moreover, cardiovascular toxicity often progresses gradually, and clinical interventions become limited once irreversible pathological changes occur (9, 10).

Consequently, early identification of high-risk individuals and implementation of preventive interventions are key strategies for improving prognosis (11, 12). Predicting the risk of cardiovascular events in breast cancer patients undergoing antineoplastic therapy enables earlier detection of cardiovascular toxicity, facilitating timely assessment and management during treatment. Cardiovascular risk prediction in breast cancer has relied heavily on traditional factors like age and hypertension (13). However, studies employing interpretable machine learning to integrate multidimensional clinical data are still lacking, particularly in China (14–16). Most studies focus on single biomarkers, fail to incorporate baseline cardiovascular status, or are constrained by small sample sizes in studies targeting long-term cardiovascular events. Based on a seven-year follow-up, Romond (17)et al. demonstrated that the addition of trastuzumab to adjuvant therapy significantly increases the risk of cardiac events. The study established an initial early scoring system based on patient age and baseline left ventricular ejection fraction (LVEF), providing an essential reference for the early warning of cardiotoxicity. Ezaz (18) et al. conducted an in-depth analysis of elderly breast cancer patients utilizing the SEER-Medicare database. The researchers developed a risk prediction model comprising seven indicators, including age, adjuvant chemotherapy regimen, coronary artery disease, atrial fibrillation, diabetes, hypertension, and renal failure, which effectively achieved cardiac risk stratification for the elderly population. Thus this study aims to develop a machine learning model to predict 1- to 3-year cardiovascular toxicity risk in breast cancer patients by integrating electronic medical records, laboratory results, and cardiac imaging. The model will incorporate short-term decline in cardiac function to assess its predictive value for long-term adverse events, enabling early warning and personalized risk management.

## Data sources and methods

### Data sources

Data for this study were obtained from the Intelligent Medical Management and Service Platform of the First Affiliated Hospital of Zhengzhou University. The platform maintains a research-ready database comprising 2,589 structured fields, including comprehensive clinical information, covers a wide range of diagnostic and follow-up data across multiple categories such as demographic characteristics, chief complaints, medical histories, imaging examinations, pathology reports, electrocardiograms (ECG), endoscopic findings, and laboratory test results. The study protocol was reviewed and approved by the Institutional Review Board (IRB) of the First Affiliated Hospital of Zhengzhou University (Approval No: 2025-KY-1562-001). All electronic medical records used in this research underwent rigorous de-identification procedures to protect patient privacy and data security protocols to prevent potential data leakage or misuse during storage and processing.

### Identification of study population

**Figure 1:**
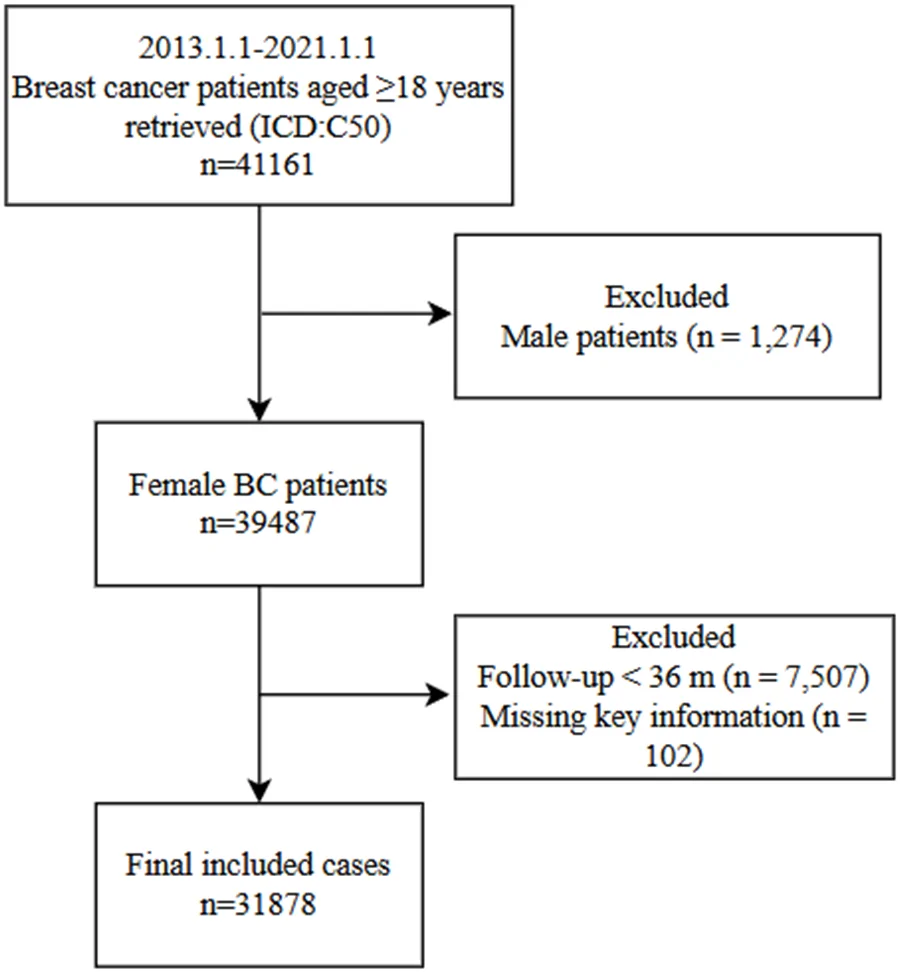
Patient flow diagram illustrating the stepwise identification of the final study cohort.

Female patients with breast cancer were identified from the Intelligent Medical Management and Service Platform of the First Affiliated Hospital of Zhengzhou University between January 1, 2013, and January 1, 2021. Case identification was based on either a diagnosis containing relevant clinical terms or the corresponding ICD-10 code (C50). Eligible patients met the following inclusion criteria: (1) age≥18 years at diagnosis; (2) pathologically confirmed breast cancer; (3) documented receipt of at least one line of antineoplastic treatment (e.g., surgery, chemotherapy, endocrine therapy) at the hospital; (4) a minimum follow-up duration of one year. Patients with missing critical information, such as primary diagnosis details or essential demographic identifiers, were excluded. The patient enrollment and exclusion process and results are shown in **Figure 1**.

### Definition of outcomes

In accordance with evidence-based criteria from the 2022 European Society of Cardiology (ESC) Guidelines on Oncocardiology, and incorporating clinical practitioner recommendations, long-term cardiovascular adverse events are defined as the following fourteen categories: (1)Heart failure; (2) Arrhythmia; (3) Cardiomyopathy; (4) Atrial fibrillation/flutter; (5) Myocardial dysfunction; (6) Ischemic heart disease/Coronary artery disease; (7) Valvular heart disease; (8) Thromboembolic disease; (9) Cerebral, pulmonary, or peripheral vascular disease; (10) Aortic aneurysm; (11) Hypertension; (12) Composite cardiovascular events (acute myocardial infarction, unstable angina, transient ischemic attack, stroke, peripheral vascular disease, hospitalization for heart failure, and death from circulatory system disease). Short-term (≤3 months) cardiovascular events were determined based on cardiac imaging and functional assessment results, encompassing objective indicators such as reduced cardiac function (left ventricular ejection fraction decrease ≥10% or absolute value <50%), valvular dysfunction, and abnormal myocardial strain. The primary endpoint was the time to the first occurrence of any cardiovascular event within 36 months of treatment initiation. The complete list of ICD-10 codes used to identify each category is provided in **Supplementary Table 1**.

### Statistical analysis methods

Continuous variables are summarized as mean (standard deviation), and categorical variables as frequency (percentage). Variables with >30% missing data were excluded. For remaining missing values, we used multiple imputation by chained equations (MICE) to create five imputed datasets, and pooled the results according to Rubin’s rules. Between-group differences in baseline characteristics were assessed using standardized mean differences (SMD).

**Figure 2:**
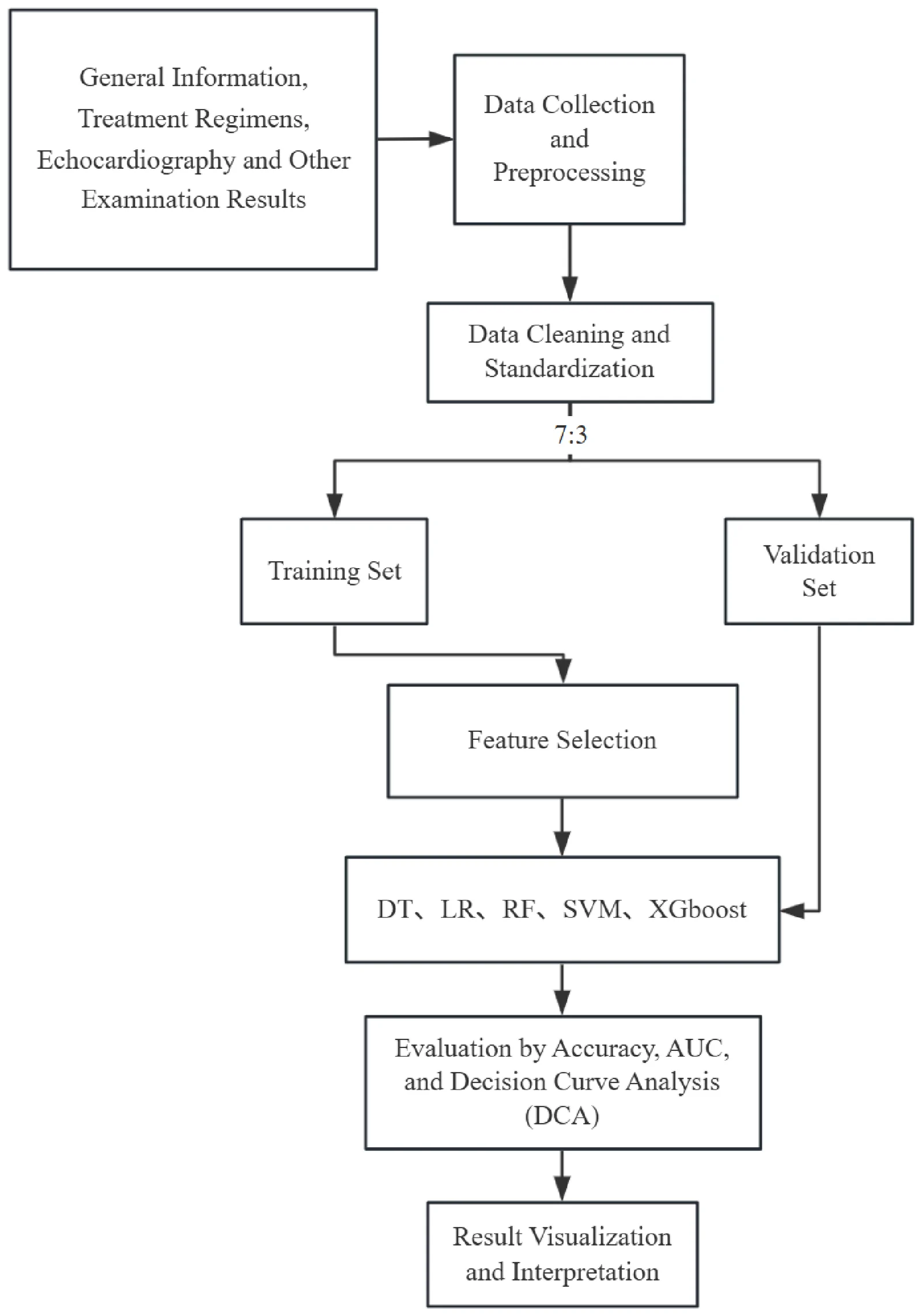
Schematic illustration of the overall analytical workflow.

To ensure clinical applicability, routinely available clinical variables at breast cancer diagnosis were included. Highly correlated variables (|r|≥0.7) identified by Spearman correlation analysis were reduced by retaining the clinically more meaningful indicators. Feature selection was further performed using least absolute shrinkage and selection operator (LASSO) regression with 10-fold cross-validation to identify the most predictive features. The dataset was randomly split into a training set (70%) and an independent test set (30%) for model development and validation. Using the refined variable set, five prediction models were developed on the training data: Logistic Regression (LR), Random Forest (RF), Extreme Gradient Boosting (XGBoost), Decision Tree (DT) and Support Vector Machine(SVM). The performance of each model was evaluated on the held-out test set using the area under the receiver operating characteristic curve (AUC-ROC). Differences in AUC between models were statistically compared using DeLong’s test. The model with the optimal performance was interpreted using SHapley Additive exPlanations (SHAP) to quantify and visualize the contribution of each feature to the predictions. The analysis was restricted to time-to-first-event; recurrent hospitalizations or subsequent distinct cardiovascular events occurring after the initial endpoint were not treated as separate outcomes. This approach avoids within-subject correlation bias arising from multiple events in the same patient. The complete processing workflow is shown in **Figure 2**.

All statistical analyses were performed using R software (version 4.3.0). A p-value < 0.05 was considered statistically significant.

## Results

### Demographic and clinical characteristics

The final study cohort comprised 31,878 breast cancer patients. Among them, 3,960 (12.4%) developed cardiovascular events and constituted the CVD group. Patients in the CVD group were significantly older than those in the non-CVD group (p < 0.001), with higher prevalence of invasive carcinoma and lymph node metastasis (all p < 0.05). Treatment patterns differed notably between groups: the CVD group received more intensive surgical and systemic therapy, and concomitant use of dexrazoxane was more common (p = 0.002). Treatment-related short-term adverse events were consistently more frequent in the CVD group, including intraoperative fluid administration, nausea/vomiting, weight loss, and body cavity effusion (all p < 0.05). Most importantly, short-term cardiac dysfunction was significantly more prevalent in the CVD group (p < 0.001) (**Table 1**).

**Table 1:** Baseline characteristics and outcomes of patients with breast cancer.

Characteristics	non-CVD group n(%)	CVD group n(%)	p-value
Total	27,918	3,960	
Demographic characteristics			
Age			<0.001
18–34	1,900 (6.8)	225 (5.7)	
35–49	11,673 (41.8)	1,377 (34.8)	
50–64	11,712 (42.0)	1,791 (45.2)	
≥65	2,6313 (9.4)	567 (14.3)	
Ethnicity			<0.001
Han Chinese	25,836 (92.5)	3,739 (94.4)	
Other	2,082 (7.5)	221 (5.6)	
Marital Status			0.043
Married	26,860 (96.2)	3,810 (96.2)	
Divorced/Widowed	632 (2.3)	111 (2.8)	
Unmarried	355 (1.3)	39 (1.0)	
Other	71 (0.3)	6 (0.2)	
Insurance Type			<0.001
New Rural Cooperative Medical Scheme	10,880 (39.0)	1,490 (37.6)	
Fully Self-funded	8,533 (30.6)	1,046 (26.4)	
Urban Basic Medical Insurance	7,025 (25.2)	1,177 (29.7)	
Other	1,480 (5.3)	247 (6.2)	
Lifestyle			
Smoking	55 (0.2)	9 (0.2)	0.835
Alcohol consumption	117 (0.4)	15 (0.4)	0.812
Tumor characteristics			
Histological type (%)			<0.001
Invasive carcinoma	24,678 (88.4)	3,588 (90.6)	
Carcinoma in situ	2,512 (9.0)	282 (7.1)	
Other	728 (2.6)	90 (2.3)	
Breast cancer location (%)			0.001
Left side	12,406 (44.4)	1,768 (44.6)	
Right side	10,488 (37.6)	1,529 (38.6)	
Both sides	5024 (18.0)	663(16.7)	
Family history of tumors	4,404 (15.8)	631 (15.9)	0.815
Lymph node metastasis	10,773 (38.6)	1,756 (44.3)	<0.001
Other metastases	3,627 (13.0)	567 (14.3)	0.022
Treatment modality			
Modified radical mastectomy	8,466 (30.3)	1,371 (34.6)	<0.001
Vacuum-assisted breast biopsy	6,133 (22.0)	1,002 (25.3)	<0.001
Breast-conserving surgery	3,043 (10.9)	453 (11.4)	0.322
Simple mastectomy	6,802 (24.4)	825 (20.8)	<0.001
Chemotherapy	18,191 (65.2)	2,830 (71.5)	<0.001
Chemotherapy regimen (%)			<0.001
Anthracycline-based regimen	10,173 (36.4)	1,656 (41.8)	
Non-anthracycline regimen	6,079 (21.8)	929 (23.5)	
Anemia management therapy	9,257 (33.2)	1,710 (43.2)	<0.001
Endocrine therapy	14,216 (50.9)	2,323 (58.7)	<0.001
Anti-angiogenic therapy	693 (2.5)	140 (3.5)	<0.001
Immunotherapy	24 (0.1)	4 (0.1)	0.990
Targeted therapy	4,624 (16.6)	837 (21.1)	<0.001
Granulocyte colony-stimulating factor (G-CSF) therapy	18,131 (64.9)	2,865 (72.3)	<0.001
Thrombopoietin therapy	5,993 (21.5)	988 (24.9)	<0.001
Traditional Chinese medicine therapy	14,293 (51.2)	2,436 (61.5)	<0.001
Combination with dexrazoxane	5,480 (19.6)	861 (21.7)	0.002
Other Clinical Characteristics			
Maximum Number of Hospitalizations (mean ± SD)	7.80 ± 6.93	10.04 ± 8.01	<0.001
Total Number of Procedures (mean ± SD)	0.81 ± 0.72	0.89 ± 0.72	<0.001
Number of Abortions (mean ± SD)	0.74 ± 1.15	0.74 ± 1.13	0.834
Intraoperative Fluid Administration	6,383 (22.9)	1,049 (26.5)	<0.001
Nausea/Vomiting	1,711 (6.1)	416 (10.5)	<0.001
Weight Loss	1,019 (3.6)	177 (4.5)	0.013
Body cavity effusion	9,294 (33.3)	1,539 (38.9)	<0.001
Decline in cardiac function	10,234 (36.7)	1,809 (45.7)	<0.001
History of Comorbidities			
Other Malignancies	1,461 (5.2)	266 (6.7)	<0.001
Cerebrovascular Disease	1,576 (5.6)	716 (18.1)	<0.001
Hepatic Disease	6,700 (24.0)	1,392 (35.2)	<0.001
Thrombosis	1,454 (5.2)	468 (11.8)	<0.001
Diabetes	2,490 (8.9)	570 (14.4)	<0.001
Renal Disease	1,576 (5.6)	420 (10.6)	<0.001
Hypertension	5,078 (18.2)	1,231 (31.1)	<0.001
Cardiac Disease	1,024 (3.7)	448 (11.3)	<0.001
Dyslipidemia	1,431 (5.1)	621 (15.7)	<0.001
Pulmonary Disease	3,017 (10.8)	771 (19.5)	<0.001
Thyroid Disorder	6,584 (23.6)	1,447 (36.5)	<0.001
Anemia	8,243 (29.5)	1,397 (35.3)	<0.001

Data are presented as n (%) or mean (standard deviation).

CVD, cardiovascular disease.

### Variable selection

**Figure 3:**
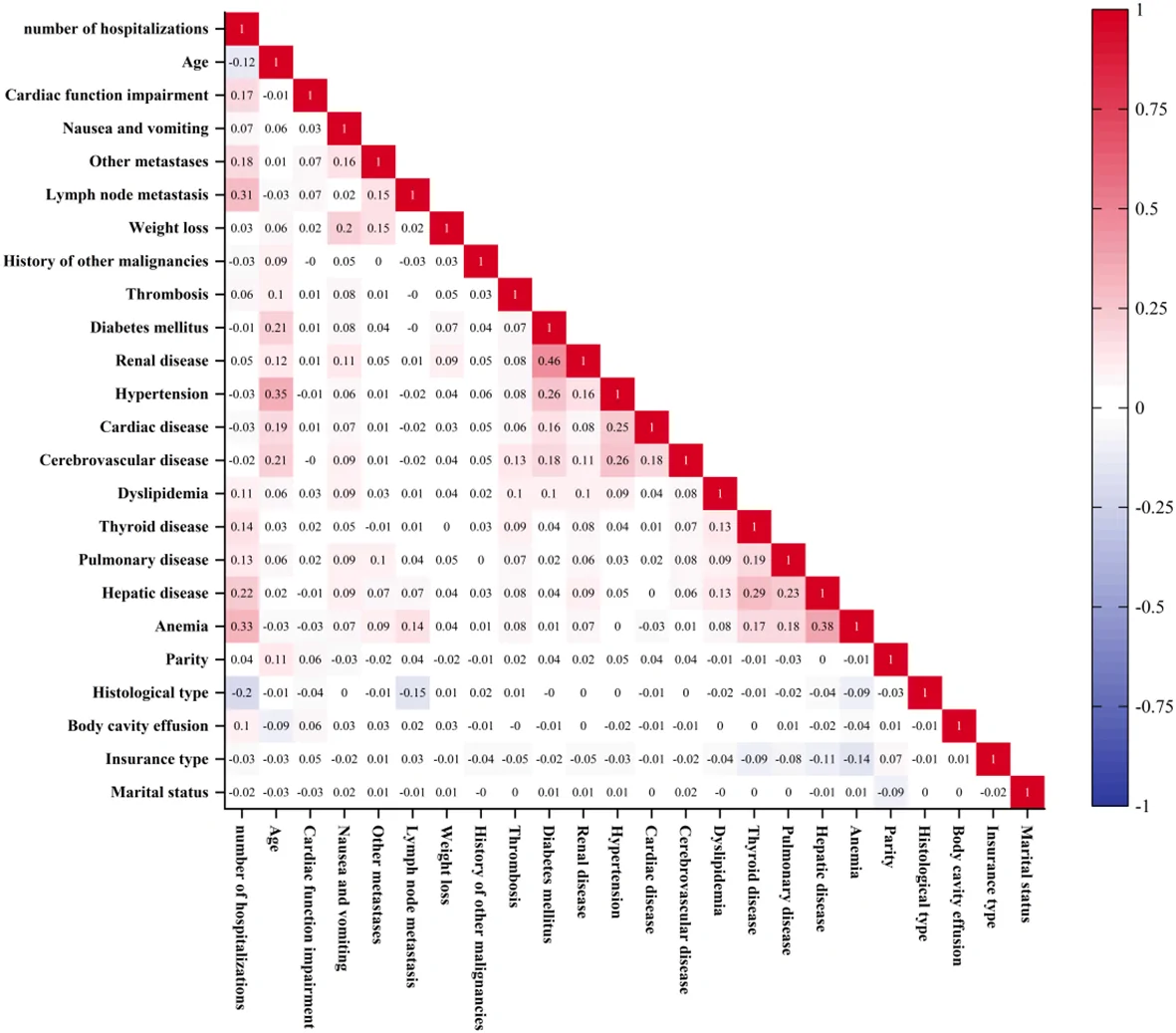
Correlation analysis results of clinical indicators.

**Figure 4:**
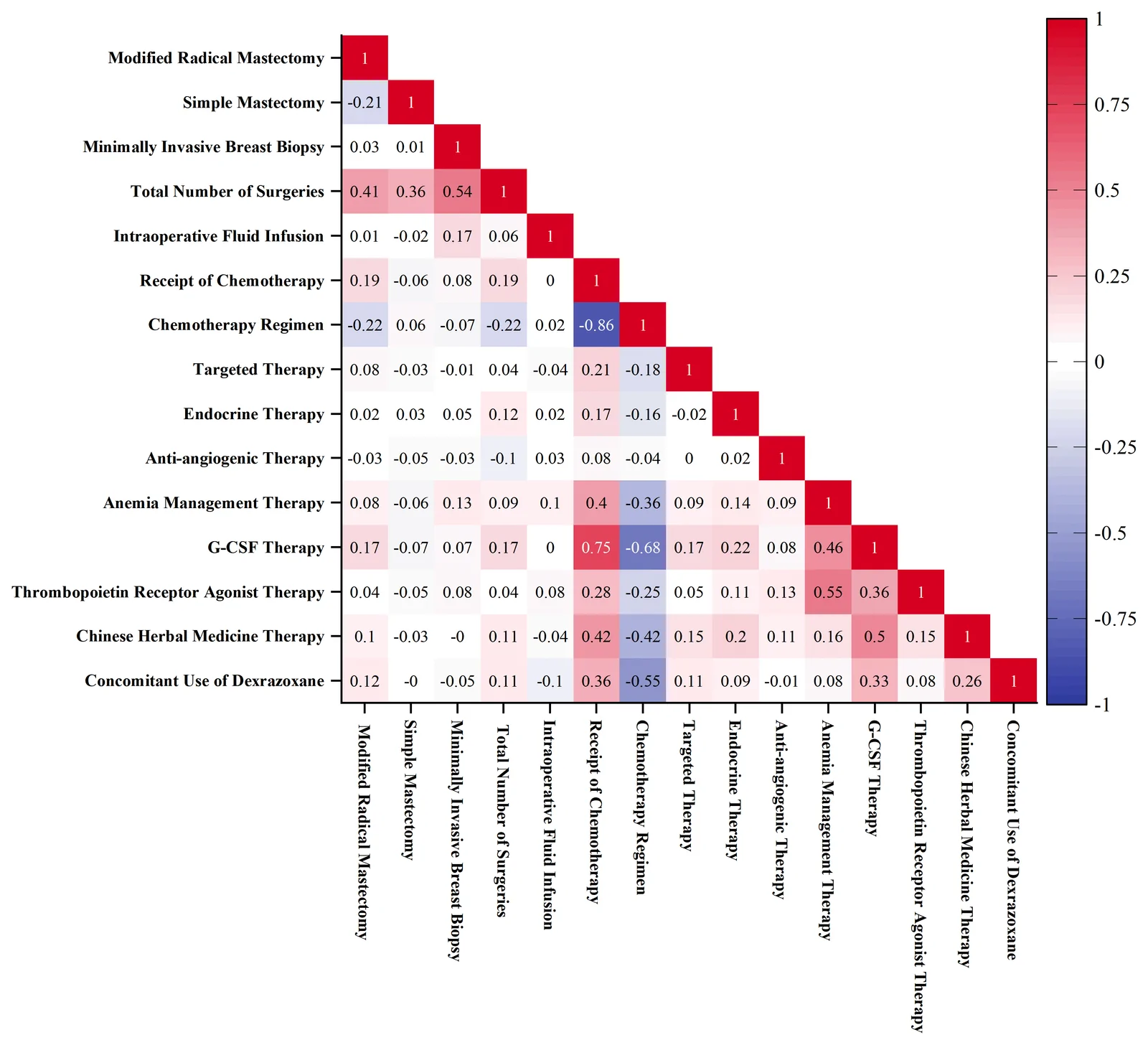
Correlation analysis results of of the treatment protocol correlation analysis.

The results of the correlation analysis between the variables are presented in **Figures 3**, **4**. Correlation analysis revealed predominantly low-to-moderate associations (|r| < 0.5) among demographic, comorbidity, and tumor characteristic variables, with no evidence of strong multicollinearity, supporting their inclusion in subsequent multivariable modeling. In contrast, several treatment-related variables showed high correlations, particularly between chemotherapy status and chemotherapy regimen (r = 0.86), and between chemotherapy status and leukocyte-raising therapy (r = 0.75; p < 0.05).

Feature selection was conducted using Least Absolute Shrinkage and Selection Operator (LASSO) regression integrated with 10-fold cross-validation. As illustrated in **Figure 3**, the coefficient paths for all 62 candidate predictors, which included clinical history, treatment regimens, and laboratory parameters, exhibited a progressive shrinkage toward zero as the penalty parameter (λ) increased. The optimal λ value was determined by minimizing the cross-validation error, which resulted in the retention of 18 variables with non-zero coefficients for subsequent multivariate modeling. The following 18 predictors were retained after LASSO regularization: Endocrine therapy, Anemia management therapy, History of cerebrovascular disease, Dyslipidemia, Decline in cardiac function, Chemotherapy regimen, Hypertension, Serum potassium level, Thyroid disease, Liver disease, Lung disease, History of cardiac disease, Age at admission,Thrombosis,Simple mastectomy,Thrombopoietin therapy,Other metastases and History of renal disease.

### Model performance and interpretation

**Figure 5:**
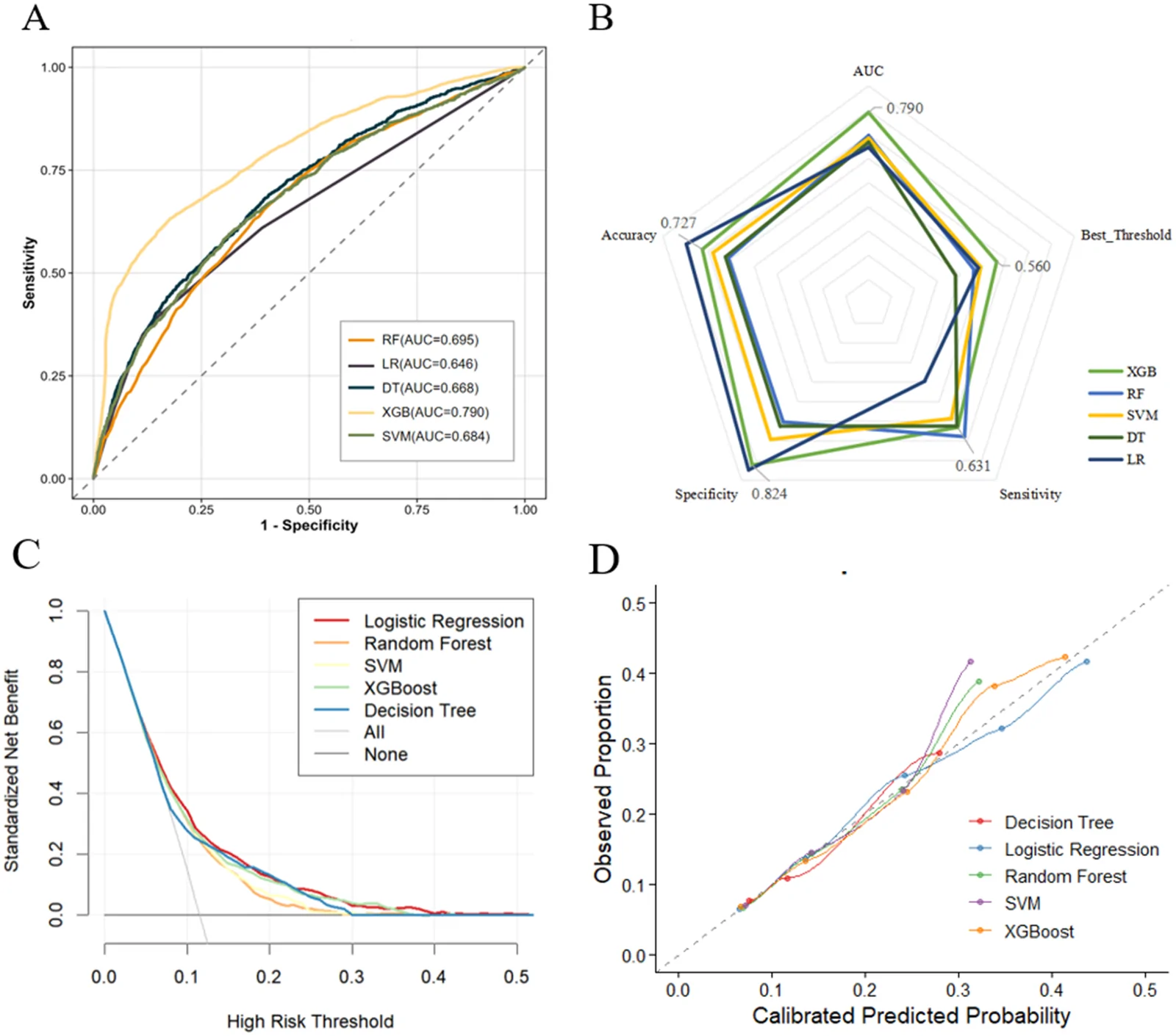
Performance comparison of the five models on the test set. **(A)** Receiver Operating Characteristic **(ROC)** Curves of Five Machine Learning Models. **(B)** Model Performance Metrics: AUC vs. Accuracy at Optimal Threshold. **(C)** Decision curve analysis **(DCA)** showing the net benefit of each model across a range of threshold probabilities. **(D)** Calibration curves of the five models.

The discriminatory performance of the developed prediction models was evaluated on an independent test set using the area under the receiver operating characteristic curve (AUC), sensitivity, and specificity. All models demonstrated predictive utility, though their performance profiles differed considerably (**Figure 5**). The Random Forest (RF) model achieved the highest sensitivity (0.679) but the lowest specificity (0.604). The Decision Tree models exhibited balanced performance. The XGBoost model attaining the highest overall discriminative ability, as reflected by an AUC of 0.790. Subsequent decision curve and calibration curve analyses consistently demonstrated the robustness of the XGBoost model, which exhibited excellent calibration and net clinical benefit among all models evaluated.

### Sensitivity analysis

**Figure 6:**
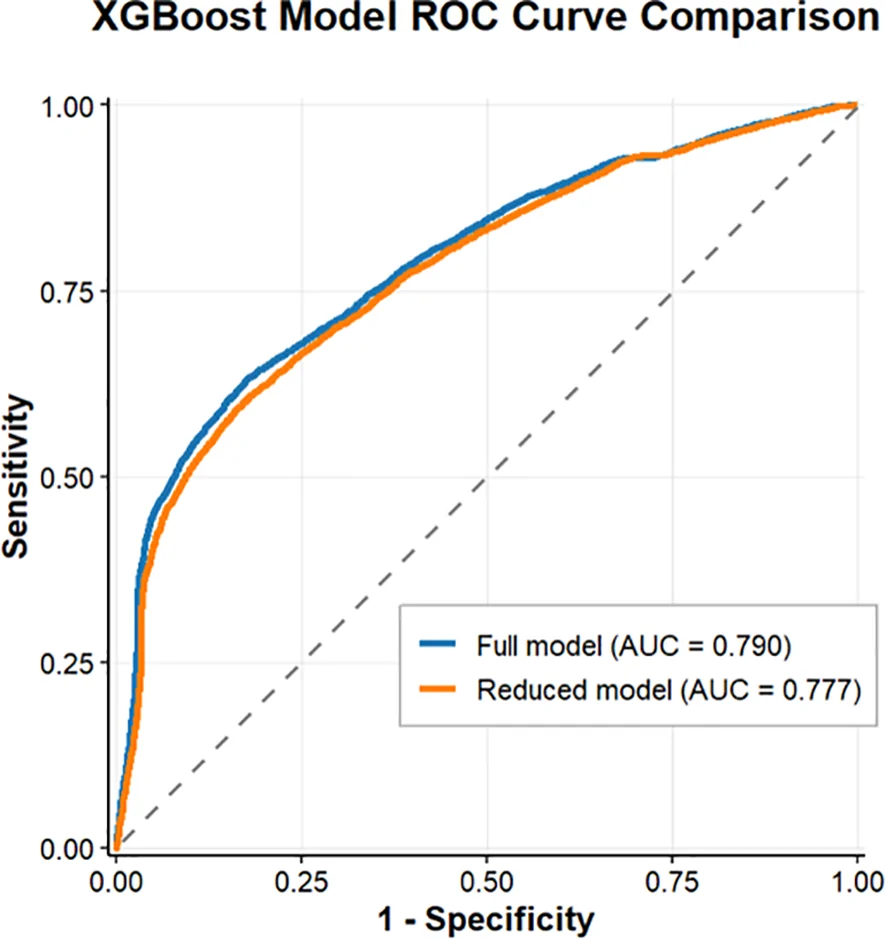
Performance comparison between models with and without prior cardiac disease.

To evaluate the robustness of the model and to assess whether its performance was overly dependent on the strong predictor of prior cardiac disease, we conducted a sensitivity analysis comparing the full model with a reduced model that excluded this variable. The results showed that the area under the curve (AUC) was 0.790 for the full model and 0.777 for the reduced model, representing an absolute difference of only 0.013 (**Figure 6**). Although statistical testing indicated that this difference was significant (P = 0.0039), such a marginal change in AUC is generally not considered clinically or practically meaningful. Therefore, it can be confirmed that while the model incorporates prior cardiac disease, it does not rely excessively on this single predictor, demonstrating good generalizability and robustness.

### Model interpretation using SHAP values

**Figure 7:**
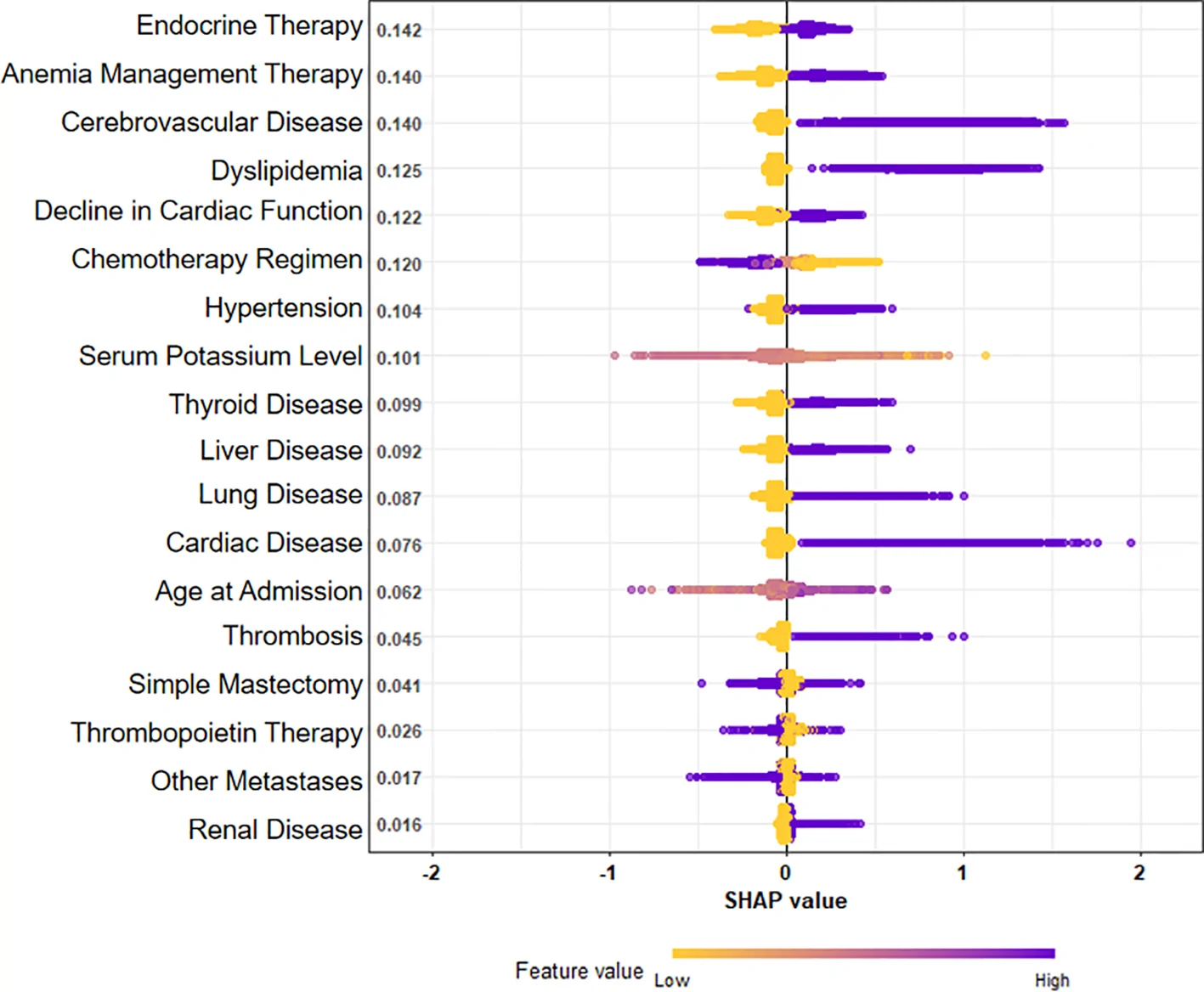
Interpretation of SHAP values for the XGBoost model.

To elucidate the decision-making mechanism of the final prediction model (XGBoost) and enhance its clinical interpretability, we performed Shapley Additive exPlanations (SHAP) analysis. The results quantified the contribution magnitude and directional influence of each predictor on model outputs (**Figure 7**). SHAP analysis identified endocrine therapy as the most influential predictor (mean |SHAP| = 0.142), followed by anemia management therapy, history of cerebrovascular disease, dyslipidemia, decline in cardiac function, chemotherapy regimen, and hypertension. Clinically, receipt of endocrine therapy, anemia management therapy, and specific chemotherapy regimens was associated with elevated cardiovascular risk. Furthermore, clinically established risk factors such as a history of cerebrovascular disease, hypertension, and dyslipidemia contributed substantially to risk prediction.

## Discussion

The development of clinical prediction models based on risk factors represents a crucial approach for achieving precision management of cardiovascular risk in cancer patients. This study leveraged real-world healthcare data to integrate multidimensional predictors and multiple machine learning algorithms, establishing and validating a composite cardiovascular event prediction model specifically for breast cancer patients. The primary translational objective was to provide clinicians with a comprehensive, generalizable, and adaptable risk stratification tool. Regarding study design, we adopted a composite cardiovascular endpoint encompassing multiple event types, consistent with internationally recognized frameworks such as the HFA-ICOS risk tool (19) and previous studies investigating major adverse cardiovascular events (4, 14). This approach enhances the clinical relevance and utility of the model by aligning with contemporary cardio-oncology practice. In predictor selection, we systematically incorporated treatment-related variables, including surgical approach, chemotherapy regimen, and supportive therapies, to fully capture the cardiovascular implications of anticancer treatments.

Our comparative analysis of multiple algorithms demonstrated that machine learning methods offer advantages in handling high-dimensional complex data, the clinical implementation has been hindered by limited interpretability of internal decision processes. To address this fundamental limitation, we implemented the SHAP framework for *post-hoc* model interpretation, which quantifies the contribution of each predictor and enables identification of clinically meaningful risk patterns.

Among the 31,878 breast cancer patients included in this study, 3,960 (12.4%) experienced at least one cardiovascular event during follow-up. Comparative analysis of baseline characteristics revealed that patients in the cardiovascular event group were generally older, had more comorbidities, exhibited greater tumor burden, and received more intensive comprehensive treatment, which is consistent with previous epidemiological reports (20, 21). Through a two-step feature selection process comprising correlation analysis and LASSO regression, we identified 18 core predictors from an initial set of 62 variables. This concise predictor set encompassed demographic characteristics, comorbidities, tumor features, treatment regimens, key short-term outcomes, and laboratory parameters. SHAP analysis not only confirmed the predictive value of these variables but also provided actionable insights for cardio-oncology practice. Endocrine therapy, anemia management therapy, and a history of cerebrovascular disease emerged as the most influential predictors, highlighting the necessity of multidisciplinary risk mitigation. The biological plausibility of the remaining predictors is well-established. Anthracycline-based chemotherapy induces direct myocardial cell damage through topoisomerase-IIβ inhibition and the generation of reactive oxygen species (22). Similarly, aromatase inhibitors used in endocrine therapy trigger metabolic alterations, such as dyslipidemia and insulin resistance, which subsequently accelerate atherosclerosis (23). The high feature importance of endocrine therapy (SHAP = 0.142) underscores that cardiovascular surveillance must extend beyond the acute chemotherapy phase. Aromatase inhibitors can induce long-term metabolic alterations, such as dyslipidemia and insulin resistance, accelerating atherosclerosis. This emphasizes the clinical need for routine metabolic monitoring and long-term cardiovascular follow-up in breast cancer survivors on prolonged endocrine therapy regimens. The prominence of anemia management therapy (SHAP = 0.140) serves as a critical clinical surrogate marker. In practice, the requirement for anemia management often reflects high-intensity chemotherapy or overall physiological frailty, while erythropoiesis-stimulating agents themselves carry intrinsic thrombotic risks. Consequently, patients requiring such supportive therapies warrant more stringent cardiovascular monitoring and careful risk-benefit assessments during treatment. The clustering of baseline chronic conditions—specifically cerebrovascular disease (SHAP = 0.140), dyslipidemia (SHAP = 0.125), and hypertension (SHAP = 0.104), highlights the compounding impact of pre-existing endothelial dysfunction. This finding strongly supports the routine implementation of comprehensive cardiovascular risk stratification prior to antitumor therapy, facilitating early cardioprotective interventions or regimen optimization for high-risk populations. Finally, short-term decline in cardiac function (SHAP = 0.122) was validated as a significant precursor to overt clinical events. This reinforces the clinical value of integrating advanced echocardiography (e.g., global longitudinal strain) and cardiac biomarkers into standard care to detect subclinical impairment, thereby allowing for the timely initiation of neurohormonal blockade to prevent irreversible heart failure.

Assessments utilizing the electronic health record frailty index (eFI) revealed a significant monotonic dose-response relationship between the degree of frailty and the incidence of adverse cardiovascular events. This association exhibited variations across racial and ethnic groups; notably, higher frailty levels were significantly correlated with an increased risk of cardiotoxicity among non-Hispanic White and Black patients (24, 25). In our systematic comparison of five algorithms, XGBoost demonstrated superior discriminative ability (AUC = 0.790) while maintaining robust performance in sensitivity analyses, confirming that machine learning approaches offer distinct advantages for analyzing structured medical data.

This study demonstrates the feasibility of developing cardiovascular risk prediction models for breast cancer patients using electronic medical record data, highlighting the potential of real-world data in cardio-oncology. Our model comprehensively covers multiple cardiovascular endpoints and shows promising clinical generalizability. However, several limitations warrant consideration. First, the single-center, regional nature of our dataset may limit the generalizability of our findings. Second, the EMR system lacked certain clinically relevant variables, including detailed tumor molecular subtypes and body mass index(BMI). Despite these limitations, our modeling strategy partially addresses these gaps. The integrated treatment variables, including chemotherapy regimens and endocrine therapy, serve as proxies for both underlying tumor biology and treatment intensity. Although our composite cardiovascular endpoint was grounded in the 2022 ESC Guidelines on Cardio-Oncology, the inherent clinical heterogeneity of the 12 constituent event categories remains a concern. Due to the relatively low incidence of certain individual events, event-specific prediction models could not be reliably developed in the current cohort. We have acknowledged this issue and suggest that future studies with larger sample sizes pursue outcome-specific analyses to further refine risk stratification. Finally, as with any retrospective study using electronic health records, the possibility of residual confounding, misclassification of outcomes based on ICD-10 codes, and unmeasured variables. Furthermore, the inclusion of metabolic comorbidities, such as hypertension and diabetes, effectively captures cardiovascular risks related to obesity and other unmeasured confounding factors. Future research should focus on several key areas: conducting multicenter, prospective external validation studies in more diverse populations; integrating tumor molecular subtyping, serial cardiac imaging parameters, and sensitive biomarkers to develop dynamic risk prediction models; In future studies, we will further utilize electronic health record (EHR) data and integrate the electronic health record-derived frailty index (eFI) to evaluate the generalizability of this predictive model across diverse clinical settings. Additionally, we will investigate the interactions among frailty, social and environmental determinants of health, and biological markers to identify factors that can further enhance the precision of risk assessments, thereby achieving more equitable and personalized risk management strategies. Finally, we choose a binary classification framework because this design prioritizes ease of clinical implementation, allowing clinicians to make binary decisions regarding the need for intensified cardiovascular surveillance. However, time-to-event analysis represents a valuable complementary approach that would make full use of censored observations and preserve all enrolled patients. Future studies with complete long-term follow-up are warranted to validate our findings using time-to-event methodology and to extend prediction to later time windows.

## Data Availability

The original contributions presented in the study are included in the article/**Supplementary Material**. Further inquiries can be directed to the corresponding authors.
