# Supplementary material for: Predicting treatment-related cardiovascular risks in breast cancer patients: development and validation of an interpretable machine learning model
Source: Front Oncol. 2026 Aug 12;16:1814219. doi: 10.3389/fonc.2026.1814219 (PMC13506277; doi:10.3389/fonc.2026.1814219)
Supplement: Supplementary file 1 [file Table1.pdf]

Supplementary Table 1. Outcome Events and Corresponding ICD-10 Codes

	Outcome Event	ICD-10 Code(s)
1	Heart failure	I50, I11.0, I13.0, I13.2
2	Cardiomyopathy	I42.0–I42.9, I43, I51.4
3	Arrhythmia	I44–I49
4	Atrial fibrillation/flutter	I48.0–I48.4, I48.91–I48.92
5	Coronary artery disease	I20-I25
6	Valvular heart disease	I34-I37, I05-I08
7	Thromboembolic	I26, I63, I74, I82
8	peripheral vascular disease	I70.0, I70.2, I70.8–I70.9, I73.8–I73.9
9	Aortic aneurysm	I71
10	Hypertension	I10-I16
11	Myocardial dysfunction	I51.89
12	Composite cardiovascular events	Including death from circulatory system disease (I00–I99), acute myocardial infarction (I21.x–I24.x), unstable angina (I20.0), transient ischaemic attack (G45.x), stroke (I60–I64), hospitalization for heart failure (I50.x), cardiac arrest/sudden cardiac death (I46), etc.

Supplementary Table 2. Brier.Score of the five models

	Brier.Score	Null.Brier.Score	Brier.Skill.Score
Logistic Regression	0.0955	0.102	0.0645
Random Forest	0.0980	0.102	0.0401
XGBoost	0.0962	0.102	0.0577
SVM	0.0979	0.102	0.0410
Decision Tree	0.0970	0.102	0.0495
